# Optimizing the World Health Organization algorithm for HIV vertical transmission risk assessment by adding maternal self-reported antiretroviral therapy adherence

**DOI:** 10.1186/s12889-022-13543-9

**Published:** 2022-07-08

**Authors:** Sheila Fernández-Luis, Maria Grazia Lain, Miquel Serna-Pascual, Sara Domínguez-Rodríguez, Louise Kuhn, Afaaf Liberty, Shaun Barnabas, Elisa Lopez-Varela, Kennedy Otwombe, Siva Danaviah, Eleni Nastouli, Paolo Palma, Nicola Cotugno, Moira Spyer, Viviana Giannuzzi, Carlo Giaquinto, Avy Violari, Mark F. Cotton, Tacilta Nhampossa, Nigel Klein, Nastassja Ramsagar, Anita Janse van Rensburg, Osee Behuhuma, Paula Vaz, Almoustapha Issiaka Maiga, Andrea Oletto, Denise Naniche, Paolo Rossi, Pablo Rojo, Alfredo Tagarro, EPIICAL Consortium, Paolo Rossi, Carlo Giaquinto, Silvia Faggion, Daniel Gomez Pena, Inger Lindfors Rossi, William James, Alessandra Nardone, Paolo Palma, Paola Zangari, Carla Paganin, Eleni Nastouli, Moira J Spyer, Anne-Genevieve Marcelin, Vincent Calvez, Pablo Rojo, Alfredo Tagarro, Sara Dominguez, Maria Angeles Munoz, Caroline Foster, Savita Pahwa, Anita De Rossi, Mark Cotton, Nigel Klein, Deborah Persaud, Rob J. De Boer, Juliane Schroeter, Adriana Ceci, Viviana Giannuzzi, Kathrine Luzuriaga, Nicolas Chomont, Nicola Cotugno, Louise Kuhn, Andrew Yates, Avy Violari, Kennedy Otwombe, Paula Vaz, Maria Grazia Lain, Elisa López-Varela, Tacilta Nhamposssa, Denise Naniche, Ofer Levy, Philip Goulder, Mathias Lichterfeld, Holly Peay, Pr Mariam Sylla, Almoustapha Maiga

**Affiliations:** 1 Centro de Investigação em Saúde de Manhiça (CISM), Bairro Cambeve, Rua 12, Distrito da Manhiça, CP 1929 Maputo, Mozambique; 2 ISGlobal, Hospital Clínic, Universitat de Barcelona, Barcelona, Spain; 3 Fundação Ariel Glaser Contra o SIDA Pediátrico, Maputo, Mozambique; 4 Pediatrics Department, Pediatric Research and Clinical Trials Unit (UPIC), Fundación para la Investigación Biomédica del Hospital 12 de Octubre, Instituto de Investigación Sanitaria Hospital 12 de Octubre (IMAS12), Madrid, Spain; 5 Gertrude H. Sergievsky Center, Vagelos College of Physicians and Surgeons, Columbia University Irving Medical Center, New York, NY USA; 6 Perinatal HIV Research Unit, Faculty of Health Sciences, University of the Witwatersrand, Johannesburg, South Africa; 7 Family Center for Research with Ubuntu, Department of Paediatrics and Child Health, Faculty of Medicine and Health Sciences, Stellenbosch University, Cape Town, South Africa; 8 School of Public Health, Faculty of Health Sciences, University of the Witwatersrand, Johannesburg, South Africa; 9 Africa Health Research Institute (AHRI), Durban, KwaZulu-Natal South Africa; 10 Great Ormond Street Institute for Child Health (GOS ICH), University College London (UCL), London, UK; 11 Research Unit in Clinical Immunology and Vaccinology, Bambino Gesu’ Children’s Hospital, 00165 Rome, Italy; 12 Chair of Pediatrics, Department of Systems Medicine, University of Rome “Tor Vergata”, 00133 Rome, Italy; 13 Fondazione per la Ricerca Farmacologica Gianni Benzi onlus, Valenzano, Italy; 14 Department of Mother and Child Health, University of Padova, Padova, Italy; 15 Instituto Nacional de Saúde (INS), Mozambique, Maputo, Mozambique; 16 Department of Medical Biology, Gabriel Toure University Hospita, Bamako, Mali; 17 Fondazione Penta ONLUS, Padova, Italy; 18 Academic Department of Pediatrics, Children’s Hospital Bambino Gesù, Rome, Italy; 19 Pediatrics Department, Hospital Universitario Infanta Sofía; Infanta Sofia University Hospital and Henares University Hospital Foundation for Biomedical Research and Innovation (FIIB HUIS HHEN), San Sebastián de los Reyes, Madrid, Spain; 20 Pediatrics Research Group, Universidad Europea de Madrid, Madrid, Spain

**Keywords:** Vertical transmission, Mother-to-child transmission, HIV-exposed infants, Paediatric HIV, Prevention of mother-to-child transmission, Enhanced post-natal prophylaxis

## Abstract

**Background:**

The World Health Organization (WHO) risk assessment algorithm for vertical transmission of HIV (VT) assumes the availability of maternal viral load (VL) result at delivery and early viral control 4 weeks after initiating antiretroviral treatment (ART). However, in many low-and-middle-income countries, VL is often unavailable and mothers’ ART adherence may be suboptimal. We evaluate the inclusion of the mothers’ self-reported adherence into the established WHO-algorithm to identify infants eligible for enhanced post-natal prophylaxis when mothers’ VL result is not available at delivery.

**Methods:**

We used data from infants with perinatal HIV infection and their mothers enrolled from May-2018 to May-2020 in Mozambique, South Africa, and Mali. We retrospectively compared the performance of the WHO-algorithm with a modified algorithm which included mothers’ adherence as an additional factor. Infants were considered at high risk if born from mothers without a VL result in the 4 weeks before delivery and with adherence <90%.

**Results:**

At delivery, 143/184(78%) women with HIV knew their status and were on ART. Only 17(12%) obtained a VL result within 4 weeks before delivery, and 13/17(76%) of them had VL ≥1000 copies/ml. From 126 women on ART without a recent VL result, 99(79%) had been on ART for over 4 weeks. 45/99(45%) women reported suboptimal (< 90%) adherence. A total of 81/184(44%) infants were classified as high risk of VT as per the WHO-algorithm. The modified algorithm including self-adherence disclosure identified 126/184(68%) high risk infants.

**Conclusions:**

In the absence of a VL result, mothers’ self-reported adherence at delivery increases the number of identified infants eligible to receive enhanced post-natal prophylaxis.

## Background

Vertical transmission (VT) of HIV remains an unacceptable 12% in many Global Plan priority countries, where 90% of the world's pregnant women are living with HIV [1]. Since implementation of Option B+ strategy, [2] antiretroviral therapy (ART) coverage among pregnant women in eastern and southern Africa increased from 84 to 92% [3]. However, the goal of eliminating new paediatric infections by 2030 [4] will likely be compromised by multiple challenges in the VT-prevention cascade, including access to adequate postnatal prophylaxis among HIV-exposed infants [1, 5–11].

VT prevention guidelines have evolved recently. Extended nevirapine (NVP) postnatal prophylaxis and dual or triple antiretroviral (ARV) prophylaxis have shown to halve VT among HIV-exposed infants compared with short courses of AZT or NVP alone [12–17]. The latest World Health Organization (WHO) guidelines recommend enhanced post-natal prophylaxis with daily zidovudine (ZDV) and NVP for the first 6 weeks of life for HIV-exposed infants at high-risk of HIV acquisition, followed by an additional 6 weeks if high-risk and breastfeeding. HIV-exposed infants at low-risk of VT should receive 4–6 weeks of prophylaxis with daily NVP (or twice-daily AZT) [18].

High-risk infants have mothers diagnosed with HIV at delivery and are either: 1.) not on ART 2.) started treatment within 4 weeks before delivery, or 3.) had a plasma viral load (VL) > 1000 copies/mL in the 4 weeks before delivering [18]. WHO designed an algorithm to assess HIV-exposed infants risk for HIV acquisition at delivery and to identify high-risk infants eligible for enhanced post-natal prophylaxis [19]. The implementation of the WHO algorithm is challenging due to the paucity of required information for risk stratification [20]. The ‘high-risk criteria’ assume VL result availability, adequate maternal adherence, and early and sustained viral suppression after 4 weeks of ART [18]. However, VL testing coverage and monitoring, especially during pregnancy, is challenging in low-and-middle-income countries (LMIC). Many countries have scaled VL testing via point of care or dried blood spot testing. However, in routine operating conditions, many patients who are tested either don’t receive results or they are extremely delayed [1, 21, 22]. Women with poor adherence or interrupted ART during pregnancy or breastfeeding have viral rebound and increased risk of VT [23–26]. In fact, peripartum viral suppression varies from 30 to 98% in different sub-Saharan African settings, and despite an estimated 92% ART coverage among pregnant women, nearly 30% of new pediatric infections in East and Southern Africa in 2018 were related to ART interruptions during pregnancy or breastfeeding [3, 10].

We retrospectively characterized post-natal prophylaxis coverage among infants living with HIV in sub-Saharan Africa and assessed whether the inclusion of readily accessible information such as mothers’ self-reported ART adherence into the WHO algorithm improved the identification of infants with perinatal HIV infection when maternal VL resulted unavailable.

## Methods

### Study population

This analysis was nested under a broader prospective cohort study (A prospective, observational, cohort, multicentre study of Early Anti-Retroviral Treatment in HIV- infected Infants: EARTH) within the EPIICAL project (Early treated Perinatally HIV-Infected individuals: Improving Children’s Actual Life Project). The EARTH study aimed to monitor clinical, virological and immunological features of HIV-positive, HIV early treated children during the first 4 years of age, in order to identify participants with excellent viral and immunological control. The EARTH study included 1) perinatally infected infants who initiated ART ≤90 days after diagnosis and 2) breastfed infants diagnosed with HIV ≤90 days of age and starting ART ≤90 days after diagnosis.

### Standard of care for PMTCT

Lifelong ART to all pregnant women living with HIV was offered at all sites. In Mozambique, HIV-exposed infants prophylaxis consisted of 6 weeks NVP to all infants until September 2019, [19, 27] and thereafter ZDV and NVP for the first 6 weeks of life, followed by NVP for 6 weeks to all infants, as all HIV-exposed infants were considered at high-risk for VT [28]. In South Africa and Mali, enhanced post-natal prophylaxis was implemented for high-risk HIV-exposed infants throughout enrollment [19].

### Data collection and analysis

The target sample size for the EARTH study was 300 children. For this analysis, we included mother-infant pairs recruited in the EARTH study from May 1st, 2018 to May 1st 2020 (*N* = 184) in 2 Mozambican sites, 3 South African sites and 1 site in Mali. Infants receiving an HIV diagnosis at the clinic were selected to participate along with their mothers. After obtaining informed consent, study personnel administered a study-specific questionnaire to the mother in a private room at the hospital, ensuring confidentiality. This questionnaire took 30 minutes on average and included information about sociodemographic characteristics, pregnancy and delivery, HIV diagnosis and care of the mother and a specific question on the number of doses missed by the mother during the month prior to the infant’s enrollment in the study. Suboptimal adherence was considered if > 10% of the ART doses were missed in the last month.

We retrospectively classified infants at high or low VT risk according to the WHO algorithm. We then modified the algorithm to include the maternal self-assessment of adherence to ART. For all women on ART for over 4 weeks and without a VL test result 4 weeks upon delivery, suboptimal and optimal adherence were defined as < 90% and ≥ 90% self-reported ART adherence during pregnancy, respectively. Infants of mothers with suboptimal adherence were considered high risk.

CompareGroups R package [29] was used to compare sociodemographic and clinical baseline information of mothers and their infants classified as HIV-exposed infants at high risk of VT according to the two algorithms, as described previously. The variables included were infant’s age and sex, maternal WHO stage, employment, marital status, education, and maternal health conditions or severe life events that arose at any later postnatal point during the study (change in employment, break-up, new partner, loss of home or moving/relocation, death in the family). We also included infant’s post-natal prophylaxis regimens: standard (≤6 weeks with ZDV or NVP) or extended (≥ 6 weeks with NVP or ZDV or with NVP plus ZDV).

The normality of continuous variables was tested using the Shapiro-Wilk test. The Mann-Whitney Wilcoxon test was used to compare non-normally distributed continuous variables. For categorical variables, absolute and relative frequencies were calculated, and Chi-squared tests or exact Fisher tests (frequencies< 5) were performed.

## Results

**Fig. 1:**
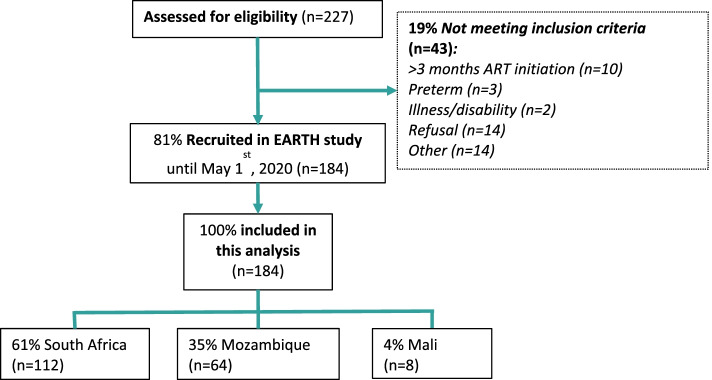
Study profile among the mother-child pairs examined for eligibility in the EARTH cohort until May 1st, 2020. Percentages are calculated over the previous parent box. ART = antiretroviral treatment

A total of 184 infants living with HIV (*n* = 96 male) and their mothers were studied, 112 (61%) from South Africa, 64 (35%) from Mozambique and 8 (4%) from Mali (Fig. 1). A total of 143 (78%) women were known HIV-positive and on ART at delivery. However, 29 women (16%) had their HIV diagnosis at delivery and were not on ART and 12 (7%) were known to be HIV-positive prior to delivery but were not on ART. Among them, only 17/143 (12%) had a VL result within 4 weeks before delivery, 13/17 (76%) of them had VL ≥1000 copies/ml. Therefore, most known HIV-positive women on ART (126/143, 88%) did not have a VL result. Of them, 99/126 (79%) had been on ART for over 4 weeks and 27/126 (21%) under 4 weeks. A total of 45/99 (45%) mothers reported suboptimal adherence to ART.

**Fig. 2:**
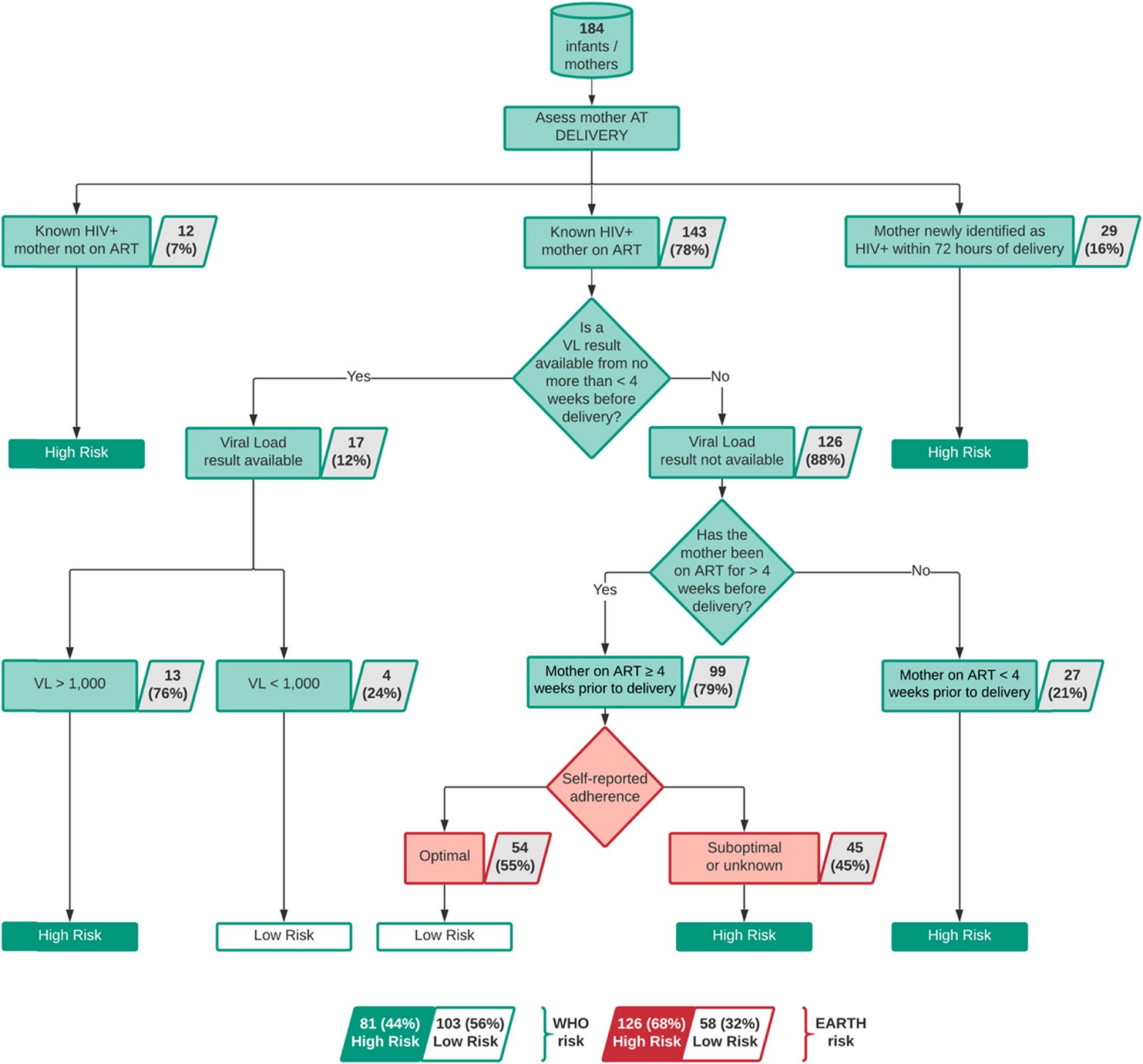
Classification of infants into high or low risk of HIV vertical transmission according to the WHO algorithm and WHO modified algorithm. The additional question was added at the last step for mothers on ART ≥4 weeks before delivery with no VL test result (in red). The WHO algorithm is described in green as well as the final classification of high-risk infants. The additional risk factor included in the modified WHO algorithm is depicted in red as well as the final classification of high-risk infants. Percentages were calculated using as denominator the number of mothers from the preceding classification step. ART = Antiretroviral treatment; VL = Viral Load

Using the WHO algorithm, 81/184 (44%) of infants with HIV were defined as high risk of VT with VL available data. Following the inclusion of the self-reported adherence risk factor applied to 99 mothers on ART greater than 4 weeks without VL test result, an additional 45 high-risk infants were identified. In total, the modified algorithm classified 126/184 (68%) infants as high risk of MTCT – assuming that infants born to mothers with suboptimal adherence are high risk (Fig. 2). The modified algorithm, identified higher number of high risk children than the WHO algorithm (*p* = 0.0001).

Among the 81 at high risk of MTCT according to WHO algorithm, 40% (32/81) received enhanced post-natal prophylaxis, 30% (24/81) standard post-natal prophylaxis, 22% (18/81) did not received any post-natal prophylaxis and 9% (7/81) did not have information about the post-natal prophylaxis.

In table 1, we compared clinical and sociodemographic characteristics that could have acted as confounding factors in the risk of VT, between participants classified as high-risk by the WHO algorithm and the additional participants classified as high risk when maternal adherence was incorporated into the algorithm. We did not find any differences in age, sex, maternal WHO status at delivery, maternal health, or social adverse events.

**Table 1:** Baseline characteristics of study participants classified as High-Risk by the WHO algorithm and the additional participants classified as high risk when maternal adherence was incorporated into the algorithm

Variable	Total	WHO Algorithm High-Risk	Additional High-Risk with the inclusion of maternal adherence	*p*-value
	*n* = 126	*n* = 81	*n* = 45	
**Age of Children at Enrollment (months)**	*n = 126*	*n = 81*	*n = 45*	
	1.57 [0.98;2.91]	1.64 [0.98;3.48]	1.38 [0.98;2.62]	0.277
Missing values	0 (0%)	0 (0%)	0 (0%)	
**Sex of Children at Birth:**	*n = 125*	*n = 80*	*n* = 45	
**Female**	55 (44.0%)	33 (41.2%)	22 (48.9%)	0.523
**Male**	70 (56.0%)	47 (58.8%)	23 (51.1%)	
Missing values	1 (0.8%)	1 (1.2%)	0 (0%)	
**Mother WHO HIV Status:**	*n = 123*	*n = 78*	*n = 45*	
**Clinical stage 1/2**	115 (93.5%)	72 (92.3%)	43 (95.6%)	1.000
**Clinical stage 3**	7 (5.69%)	5 (6.4%)	2 (4.4%)	
**Clinical stage 4**	1 (0.81%)	1 (1.28%)	0 (0.00%)	
Missing values	3 (2.4%)	3 (3.7%)	0 (0%)	
**Mother Severe Life Events / Health Issues:**	*n = 115*	*n = 74*	*n = 41*	
**No**	62 (53.9%)	41 (55.4%)	21 (51.2%)	0.813
**Yes**	53 (46.1%)	33 (44.6%)	20 (48.8%)	
Missing values	11 (8.7%)	7 (8.6%)	4 (8.9%)	
**Mother Social Adverse Events:**	*n = 126*	*n = 81*	*n = 45*	
**No**	5 (3.97%)	3 (3.7%)	2 (4.4%)	1.000
**Yes**	121 (96.0%)	78 (96.3%)	43 (95.6%)	
Missing values	0 (0%)	0 (0%)	0 (0%)	
**Mother Employment Status**	*n = 123*	*n = 79*	*n = 44*	
**Employed**	14 (11.4%)	10 (12.7%)	4 (9.1%)	0.763
**Unemployed**	109 (88.6%)	69 (87.3%)	40 (90.9%)	
Missing values	3 (2.3%)	2 (2.5%)	1 (2.2%)	
**Mother Marital Status**	*n = 126*	*n = 81*	*n = 45*	
**Single**	76 (60.3%)	49 (60.5%)	27 (60.0%)	1.000
**Married**	47 (37.3%)	30 (37.0%)	17 (37.8%)	
**In a relationship, not cohabiting**	3 (2.38%)	2 (2.47%)	1 (2.22%)	
Missing values	0 (0%)	0 (0%)	0 (0%)	
**Mother Education**	*n = 126*	*n = 81*	*n = 45*	
**No school**	3 (2.38%)	2 (2.47%)	1 (2.22%)	0.850
**Primary**	44 (34.9%)	26 (32.1%)	18 (40.0%)	
**Secondary**	74 (58.7%)	49 (60.5%)	25 (55.6%)	
**University**	5 (3.97%)	4 (4.94%)	1 (2.22%)	
Missing values	0 (0%)	0 (0%)	0 (0%)	

Continuous variables summarized as: median [IQR], and categorical variables as: n (% of subjects within classification group, excluding missing values). The first row of each section shows the number of subjects without missing corresponding information, and last row number and percentage of missing values compared to the total number of subjects. Mother Severe Life Events: Any during the time of the study, including change in employment, separation or relationship break-up, new partner, loss of home or move, death in the family, other. Mother Health Issues: Any during the time of study. Mother Employment status, Marital status and Education asked at enrolment. Mother Social Adverse Events: Unemployed, single status or did not attend high school/university (asked at enrollment). Standard Post-natal prophylaxis: < 6 weeks with one of Zidovudine (AZT) or Nevirapine (NVP)

## Discussion

In our study, nearly 90% of mothers of infants diagnosed with HIV did not have a VL result at delivery; a result on which the WHO algorithm relies. When applied in our cohort, the WHO algorithm identified 44% of the infected infants as high-risk for VT. When we added mothers’ self-reported ART adherence to the WHO algorithm, the proportion of high-risk infants increased to 68%, suggesting that suboptimal adherence is associated with high risk. This modification of the algorithm increased the number of infected infants being classified as high-risk and eligible for enhanced post-natal prophylaxis.

This study also showed that information is often missing when assessing VT risk assessment according to the WHO algorithm, which jeopardizes early prescription of enhanced post-natal prophylaxis [15]. Only 12% of mothers on ART had a VL result 4 weeks prior to delivery. Similarly, a cross-sectional study of programmatic data in Zimbabwe revealed that the risk of MTCT using the WHO algorithm could not be determined in 90% of HIV-exposed infants due to lack of data [20]. A more recent study of 2080 HIV-exposed infants from Zimbabwe showed that 80% of mothers did not have a VL result between 28 weeks gestation to delivery [30]. Therefore, other factors should be considered to establish the risk of VT. Our results suggest that self-reported maternal adherence to ART could be an additional clinical factor to be considered in order to improve the algorithm to assess the risk of VT in the absence of VL. We also evaluated other socio-clinical factors that could potentially affect the risk of MTCT, such as maternal WHO stage, level of education, employment status or adverse social events between the two algorithms. We didn’t find any difference among the participants newly classified as high risk of VT according to maternal ART adherence and the participants classified by the WHO algorithm as high risk of VT. However, further studies evaluating additional socio-clinical variables could inform more targeted algorithms to improve prevention of VT.

ART duration over 4 weeks, as incorporated in the algorithm, does not justify the assumption of viral suppression or the low VT risk. Although many countries have recently changed from EFV-based maternal ART to Dolutegravir, which has been shown to have superior early virologic suppression [31], adherence remains a paramount. Poor maternal adherence or interrupted ART during pregnancy and breastfeeding may cause high VL and subsequent increased transmission risk [23–26]. As such, infants are misclassified as ‘low-risk’ and do not receive appropriate e-PNP. The use of point-of-care devices for VL may be used at delivery and provide results in under 90 minutes, however, these devices are not widely available in LMIC [32]. Modification of the existing WHO risk evaluation algorithm to include a maternal self-reported adherence may significantly benefit countries without a consolidated laboratory network capacity to ensure optimal VL monitoring. Investigating the mothers’ self-reported ART adherence at delivery is an easy and low-cost intervention to guide nurses in identifying high-risk infants eligible for enhanced post-natal prophylaxis in the absence of VL result.

In our cohort, 45% of mothers on ART for over 4 weeks without a VL result reported sub-optimal adherence. The actual proportion might be higher, since self-reported adherence is a reliable method with low cost and good specificity [33] widely used in clinical practice, but tends to overestimate adherence behavior compared with other methods [9]. Low self-reported adherence during pregnancy and breastfeeding is associated with viremia and virological treatment failure [34, 35]. The high number of women who self-disclosed ART-adherence difficulties in our study suggests that in the absence of VL result, time on ART is not enough to evaluate VT risk. Effective interventions to support adherence during pregnancy must be considered.

Our findings showed that 30% of HIV-exposed infants at high-risk of HIV infection according to WHO algorithm had no access to enhanced post-natal prophylaxis, which can be partially due to the recent guideline implementation in Mozambique [28]. A study in Zimbabwe also showed low enhanced post-natal prophylaxis coverage rates among HIV-exposed infants [20]. We also found a high proportion (17%) of HIV-exposed infants who alarmingly did not receive any PNP at all. Further studies should evaluate enhanced post-natal prophylaxis compliance and algorithm feasibility in the clinical setting, including HIV-exposed children at high risk of VT who never become infected. Nevertheless, our results suggest that besides modifying the WHO algorithm and training health staff on its correct application, further efforts are needed to ensure access to timely maternal VL testing and infant enhanced post-natal prophylaxis.

Our results have implications for policy, practice and public health. This study provided a comprehensive evaluation of the WHO risk assessment algorithm for VT. On one hand, we identified that the coverage of enhanced postnatal prophylaxis among infants with high risk of VT transmission was not optimal. On the other hand, our results suggested that adding maternal adherence to ART could increase the coverage of enhanced postnatal prophylaxis in high-risk infants, thus reinforcing WHO recommendations in clinical practice. The application of this simple measure would be especially beneficial in settings with less structural capacity to obtain viral load results. It would allow the allocation of limited resources towards prevention measures for children at high risk and inform interventions to improve VT prevention.

This study has several limitations. First, we included only infants confirmed to have HIV infection, and retrospectively reviewed their VT risk classification. For this reason, we could not calculate the specificity of the two algorithms. Second, the mothers’ ART adherence was self-reported at recruitment and recall bias, as well as social desirability bias, may have led to over-reporting of good adherence. We defined 1 month prior to study enrollment as the time interval for observing the number of missed doses in order to improve the validity of past reporting. We based this on the fact that a shorter time frame allows the respondent to more easily recall an event rather than having to recall a behavior over a large period of time. However, further studies evaluating a standardized method and definition of self-reported adherence would be important to generalize our results on ART adherence and validate the inclusion of the self-reported adherence to ART in the algorithm in other contexts. Third, we didn’t find difference in the confounders factors analyzed, however other potential confounding factors such as type of infant feeding, home delivery or maternal drug resistance have not been accounted for. Fourth, the relatively small sample size can compromise the generalizability of the results. Further studies with larger sample size are needed to validate this results in populations from different settings. 

In conclusion, incorporation of maternal self-reported adherence in the WHO algorithm for VT risk assessment improves the identification of infants eligible for enhanced post-natal prophylaxis and should be considered in the algorithm. A study including HIV-exposed uninfected infants is needed to assess whether the modified WHO algorithm adequately identifies infants who do not need enhanced post-natal prophylaxis and remain HIV-free after breastfeeding.

## Data Availability

The datasets used and/or analyzed during the current study are available from the corresponding author on reasonable request.
